# YAP promotes the malignancy of endometrial cancer cells via regulation of IL-6 and IL-11

**DOI:** 10.1186/s10020-019-0103-4

**Published:** 2019-07-12

**Authors:** Jing Wang, Tiefang Song, Suiyang Zhou, Xianchao Kong

**Affiliations:** Department of Gynecology and Obstetrics, The 2nd Affiliated Hospital of Harbin Medical University, NO. 246, Xuefu Road, Nangang District, Harbin, 150081 Heilongjiang People’s Republic of China

**Keywords:** YAP, Endometrial Cancer, IL-6, IL-11, Proliferation

## Abstract

**Background:**

Emerging evidence shows that Hippo signal pathways can regulate the progression of various cancer. While the roles of Yes-associated protein (YAP), the key transducer of Hippo signals, in the development of endometrial cancer (EC) are rarely investigated.

**Methods:**

The expression of YAP in endometrial cancer cells and tissues was measured. Its roles in proliferation and expression of interleukins (ILs) were investigated by use of its specific siRNA or inhibitor (verteporfin, VP).

**Results:**

YAP was upregulated in endometrial cancer cells and tissues. Knockdown of YAP or VP can suppress the proliferation while increase its chemo-sensitivity of EC cells. We found that targeted inhibition of YAP can decrease the expression of interleukin-6 (IL-6) and IL-11 in EC cells. Recombinant IL-6 or IL-11 can attenuate si-YAP suppressed proliferation of EC cells. Chromatin immunoprecipitation (ChIP) assay suggested that YAP can directly bind with the promoter of IL-6 and induce its transcription. As to IL-11, inhibitor of NF-κB (BAY 11–7082) can significantly down regulate the mRNA expression of IL-11. Over expression of p65 abolished si-YAP suppressed transcription of IL-11. It suggested that NF-κB was involved in the YAP regulated expression of IL-11.

**Conclusions:**

YAP can regulate the proliferation and progression of EC cells. It suggested that targeted inhibition of YAP might be a potent potential approach for EC therapy.

## Introduction

Endometrial cancer (EC) is the most common gynecological cancer in developed countries (Dizon, 2010), with about 50,327 deaths occurring worldwide each year (Siegel et al., 2016). Further, the incidence and mortality rate are still increasing in the developed and developing countries (Rauh-Hain and Del Carmen, 2010). The 5-year overall survival ranges from 74 to 91% in patients without metastatic disease (Colombo et al., 2013). However, for EC patients with metastasis, treatment failure is still high due to the loss of opportunity for surgery. In advance stages of EC patients, the growth and systemic metastasis lead to patient morbidity and mortality (Huang et al., 2014). Previous studies indicated that some proteins are variated in the EC tissues and cells such as *PTEN*, *KRAS*, *CTNNB1*, *PIK3CA* and *FGFR2* (Tsujiura et al., 2014). Therefore, one major challenge for EC treatment is to develop efficiency approaches to block the signals essential for the progression of EC cells.

As the key downstream effector in the Hippo signaling cascade, the Yes-associated protein (YAP) is a major contributor to cancer pathophysiology (Pan, 2010; Zhao et al., 2007). The Hippo signals are composed of mammalian Ste20-like kinases 1/2 (MST1/2) and large tumor suppressor 1/2 (LATS1/2), YAP and its paralog TAZ (Liu et al., 2010). After activation of Hippo signals, MST1/2 is phosphorylated and then activates LATS1/2 (Real et al., 2018). The activation of Lats1/2 can phosphorylate YAP and subsequent promote proteasome mediated degradation (Zhao et al., 2010). Dephosphorylation of YAP can allow YAP to translocate into the nucleus, bind with its transcriptional co-activator TEAD, and increase the transcription of many oncogenes (Zhao et al., 2008). Although many studies indicated that YAP functions as an oncogene in most cancers (Zhao et al., 2010), the roles and related mechanisms of YAP on the progression of EC remain unclear. Recently studies revealed that increased nuclear YAP expression was significantly associated with higher grade, stage, lympho-vascular space invasion, postoperative recurrence/metastasis and overall survival in estrogen mediated EC patients (Tsujiura et al., 2014). It suggested that YAP may also trigger the progression of EC via unknown mechanisms.

The present study investigated the potential roles and related mechanisms of YAP in the progression of EC. Our data showed that YAP is upregulated in EC cells and tissues. Knockdown of YAP or its inhibitor verteporfin can suppress the proliferation and increase the chemo-sensitivity of endometrial cancer cells. The upregulation of interleukin-6 (IL-6) and IL-11 is essential for YAP induced malignancy of EC cells.

## Materials and methods

### Chemicals and reagents

The doxorubicin (Dox) and other chemicals/inhibitors were purchased from Sigma-Aldrich (St. Louis, MO, USA). Human recombinant IL-6 and IL11 were obtained from R&D Systems (Sydney, Australia). Scrambled control siRNA oligonucleotide (si-NC) or siRNA for YAP were purchased from Invitrogen (Life Technologies, Grand Island, NY, USA). Primary antibodies and horseradish peroxidase (HRP)-conjugated secondary antibody were purchased from the Cells Signaling Technology (Danvers, MA, USA).

### Cell culture and transfection

The human EC Ishikawa, RL95–2, HEC1A, AN3CA and KLE cells and the human endometrial cell line endometrial stromal cell (ESC) were purchased from the Cell Bank of the Chinese Academy of Sciences, Shanghai, China. After confirmed by short tandem repeat profiling, cells were cultured in medium containing 10% (v/v) FBS (Scientifix, Cheltenham, VIC, Australia) and 1% (V/V) penicillin-streptomycin solution (Sigma, St Louis, MO, USA). All cells were routinely tested as free from mycoplasma contamination. In order to knock down the expression of YAP, siRNA specific for YAP (5’GCCAGUACUGAUGCAGGUATT3’, Shanghai GenePharma Co. Ltd., Shanghai, China) was used to transfect cells by using Lipofectamine 2000 (Thermo Fisher Scientific, Waltham, MA, USA) according to the manufacturer’s instruction. Similarly, the pcDNA (vector control) and pcDNA/p65 plasmids were also transfected by use of Lipofectamine 2000.

### RNA preparation and quantitative real time RT-PCR (qRT-PCR)

Total RNAs were isolated by use of TriReagent (Sigma-Aldrich) and further purified by use of the DNA free kit (Ambion) according to the manufacturer’s instructions. The cDNA was synthesized using Superscript III reverse transcriptase (Invitrogen) and 500 ng of total RNA. Then, qRT-PCR was performed on the Bio-Rad System (Bio-Rad Laboratories Inc., Hercules, CA, USA) using 2× Fast-Start SYBR green master mix and the following primers: YAP, forward: 5′- TAGCCCTGCGTAGCCAGTTA − 3′, reverse: 5′- TCATGCTTAGTCCACTGTCTGT -3′; IL-1β, forward: 5′- ATGATGGCTTATTACAGTGGC − 3′, reverse: 5′- GTCGGAGATTCGTAGCTGGA -3′; IL-6, forward: 5′- ACTCACCTCTTCAGAACGAATTG − 3′, reverse: 5′- CCATCTTTGGAAGGTTCAGGTTG -3′; IL-8, forward: 5′- GAG AGT GAT TGA GAG TGG ACC AC − 3′, reverse: 5′- CAC AAC CCT CTG CAC CCA GTT T -3′; IL-10, forward: 5′- TCT CCG AGA TGC CTT CAG CAG A − 3′, reverse: 5′- TCA GAC AAG GCT TGG CAA CCC A -3′; IL-11, forward: 5′- GCGCTGTTCTCCTAACCCG-3′, reverse: 5′- GAGTCCAGACTGTGATCTCCG-3′; TNF-α, forward: 5′- CTC TTC TGC CTG CTG CAC TTT G − 3′, reverse: 5′- ATG GGC TAC AGG CTT GTC ACT C − 3′; p65, forward: 5′- GTGGGGACTACGACCTGAATG − 3′, reverse: 5′- GGGGCACGATTGTCAAAGATG -3′; GAPDH, forward: 5′-GGAGCGAGATCCCTCCAAAAT-3′, reverse: 5′-GGCTGTTGTCATACTTCTCATGG-3′. GAPDH was used as the internal control for normalization. The 2^−ΔΔCT^ method was used to quantify gene expression.

### Western blot analysis

Cells or tissues were lysed by use of lysis buffer containing the protease inhibitor (2 μl/ml; ThermoScientific, Waltham, MA, USA). Then total 20 μg proteins were separated by use of a 10% SDS-PAGE gel. The proteins were transferred to polyvinylidene fluoride (PVDF) membranes and incubated with the primary antibodies overnight at 4 °C. After washed three times, membranes were incubated with the secondary antibody, exposed to an enhanced chemiluminescence (ECL) western blot by use of ECL system (GE Healthcare Life Sciences), and quantified using Bio-Rad Quantity One 1-D software.

### Patient samples

According to the permission of Ethical Committee in our hospital, seven paired tumor tissues and adjacent normal tissues were collected during July 2015 to June 2017. Informed consent was obtained from each patient. The samples were stored at − 80 °C immediately after surgery. The expression of YAP was measured by use of western blot analysis.

### Cell proliferation assay

The cell proliferation was analyzed by use of the Wst-1 assay according to the previous study (Lay et al., 2012). Briefly, cells (5, 000 per well) were seeded into 96-well plates. After treatment, cells were incubated with Wst-1 dye (1:10; Roche Applied Science) for 4 h at 37 °C. The absorbance at 450 nm was measured with a Wallac Envision 2103 plate reader (Perkin Elmer).

### Enzyme-linked immunoassays (ELISAs)

The expression of IL-6 and IL-11 in medium was measured by ELSIA by use of kits according to the manufacturer’s protocol (eBioscience, USA). The absorbance at 450 nm was measured with a Wallac Envision 2103 plate reader (Perkin Elmer).

### Chromatin immunoprecipitation (ChIP) PCR

ChIP assays were performed with an Agarose ChIP Kit (Thermo Scientific) according to the manufacturer’s instructions. Briefly, after treatment as indicated conditions, cells were crosslinked and lysed. The DNA was extracted and sonicated to shear DNA into fragments of 500–1000 bp in length. The DNA/protein complexes were precipitated by antibody of YAP or IgG (ab171870, Abcam), and then incubated with Protein A/ G agarose beads for 2 h. The abundance of IL-6 or IL-11 promoter was analyzed by qPCR using primers as follows: IL-6, 5′-ACCCTCACCCTCCAACAAAG-3′ and 5′ -GCAGAATGAGCCTCAGACATC-3′; IL-11, 5′- CTTTGCTTCTCTGGTGTGTC − 3′ and 5′ - CTGGTGAGGTCATTGGCGT − 3′.

### Statistical analysis

All results were stated as mean ± standard deviation (SD). The data analysis was performed by use of GraphPad Prism 5 (GraphPad Software, San Diego, CA, USA) for Windows. Statistical comparison was performed using the Student’s *t* test for two groups. ANOVA analysis with Tukey’s multiple comparison test was also used to compare three or more groups. A *p*-value of < 0.05 was considered statistically significantly different between groups.

## Results

### The expression of YAP is upregulated in EC cells and tissues

**Fig. 1:**
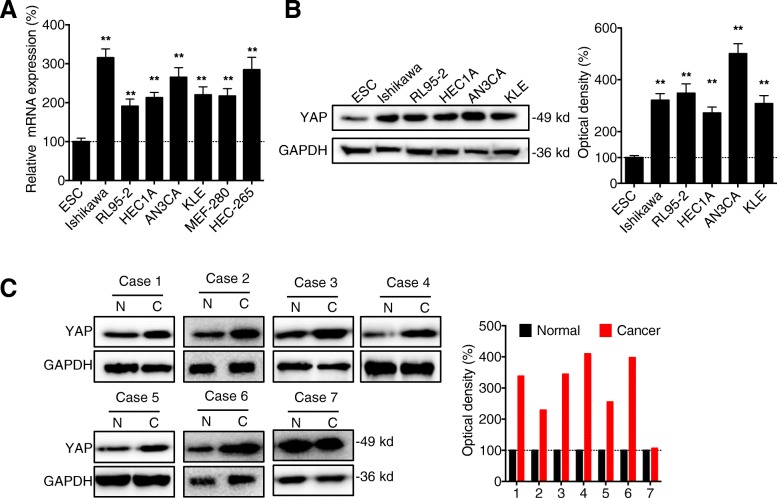
*The expression of YAP is upregulated in EC cells and tissues*. (**a**) The mRNA expression of YAP in human EC, ESC, and non-transformed endometrial cell line of epithelial origin such as MEF-280 and HEC-265 cells were measured by qRT-PCR; (**b**) The protein expression of YAP in human EC and ESC cells was measured by western blot analysis (left) and quantitatively analyzed (right); (**c**) The protein expression of YAP in seven cases of EC tissue and paired adjacent normal tissues was measured by western blot analysis (left) and quantitatively analyzed (right). Data are presented as means ± SD of three independent experiments. ** *p* < 0.01 compared with control

Firstly, the expression of YAP was measured in various EC cells and the human endometrial cell line endometrial stromal cell (ESC). Our data showed that the mRNA expression of YAP in all tested EC cell lines including Ishikawa, RL95–2, HEC1A, AN3CA, and KLE and non-transformed endometrial cell line of epithelial origin such as MEF-280 and HEC-265 cells were significantly greater than that in the ESC (Fig. 1 a). This was confirmed by western blot analysis that protein expression of YAP in EC cells was greater than that in ESC (Fig. 1 b). We further evaluated the expression of YAP in seven collected human EC tissues and the paired normal adjacent tissues. Western blot analysis showed that the expression of YAP was increased in 85.7% (6/7) human EC tissues (Fig. 1 c). These data showed that the expression of YAP is upregulated in EC cells and tissues.

### Targeted inhibition of YAP can suppress the proliferation and increase the chemosensitivity of EC cells

**Fig. 2:**
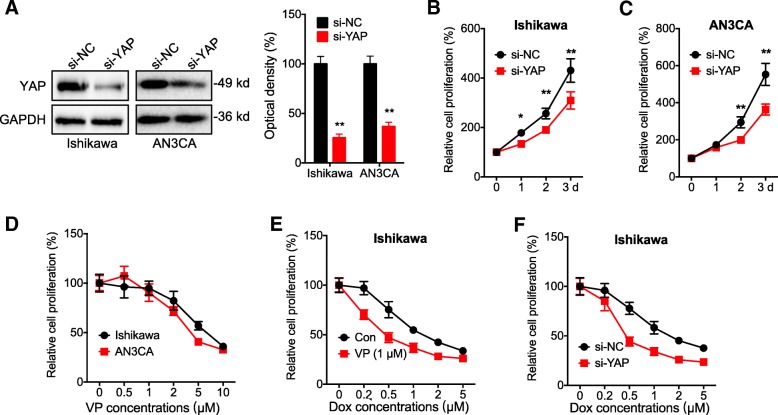
*Targeted inhibition of YAP can suppress the proliferation and increase the chemosensitivity of EC cells****.*** (**a**) Cells were transfected with siRNA negative control (si-NC) or si-YAP for 24 h, the expression of YAP was measured by western blot analysis (left) and quantitatively analyzed (right); Ishikawa (**b**) or AN3CA (**c**) cells were transfected with si-NC or si-YAP for the indicated time periods, the cell proliferation was measured by CCK-8 kit; (**d**) Ishikawa or AN3CA cells were treated with increasing concentrations of VP for 48 h, the cell proliferation was measured by CCK-8 kit; (**e**) Ishikawa cells were pre-treated with or without 1 μM of VP and then further treated with increasing concentrations of Dox for 48 h, the cell proliferation was measured by CCK-8 kit; (F) Ishikawa cells were pre-transfected with si-NC or si-YAP for 12 h and then further treated with increasing concentrations of Dox for 48 h, the cell proliferation was measured by CCK-8 kit. Data are presented as means ± SD of three independent experiments. *p < 0.05, ** *p* < 0.01 compared with control

Ishikawa and AN3CA cells were used for the next studies due to the relative high levels of YAP and they have been widely used for EC study. To investigate the potential roles of YAP in cancer progression, we knocked down the expression of YAP by use of its specific siRNA (Fig. 2 a). Our data showed that knockdown of YAP can significantly decrease the proliferation of both Ishikawa (Fig. 2 b) and AN3CA (Fig. 2 C) cells. Further, verteporfin (VP), an inhibitor of the interaction of YAP with TEAD, can also significantly inhibit the proliferation of both Ishikawa and AN3CA cells via a concentration dependent manner (Fig. 2 d). Further, 1 μM of VP, which had no significant effect on the cell proliferation according to Fig. 2 d, colonization assay (data not shown), and previous studies (Hsu et al., 2016; Ma et al., 2016; Wang et al., 2016), can increase the sensitivity of Ishikawa cells to the treatment of Doxorubicin (Dox) (Fig. 2 e). Consistently, si-YAP also increased the Dox sensitivity of Ishikawa cells (Fig. 2 F). These data suggested that targeted inhibition of YAP can suppress the proliferation and increase the chemosensitivity of EC cells.

### YAP can regulate the expression of IL-6 and IL-11 in EC cells

**Fig. 3:**
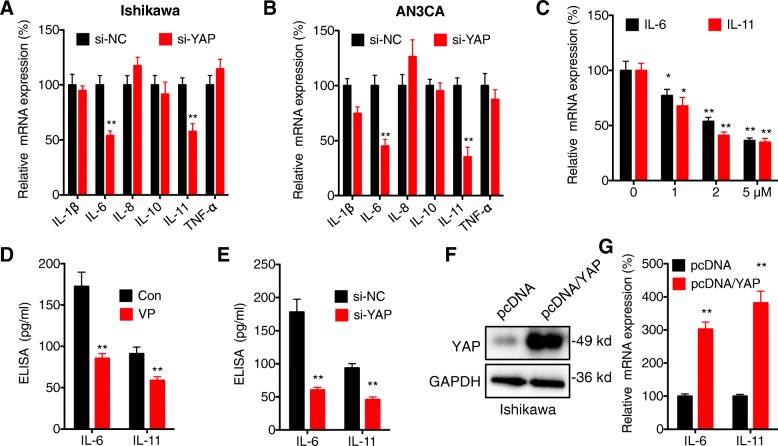
*YAP can regulate the expression of IL-6 and IL-11 in EC cells****.*** Ishikawa (**a**) or AN3CA (**b**) cells were transfected with si-NC or si-YAP for 24 h, the mRNA expression of cytokines was measured by qRT-PCR; (**c**) Ishikawa cells were treated with increasing concentrations of VP for 24 h, the mRNA expression of cytokines was measured by qRT-PCR; (**d**) Ishikawa cells were treated with or without 1 μM of VP for 24 h, the expression of cytokines was measured by ELISA; (**e**) Ishikawa cells were transfected with si-NC or si-YAP for 24 h, the expression of cytokine was measured by ELISA; Ishikawa cells were transfected with pcDNA vector or pcDNA/YAP for 24 h, (**f**) the protein expression of YAP was tested by western blot, (**g**) the mRNA expression of IL-6 and IL-11 was tested by qRT-PCR. *p < 0.05, ** *p* < 0.01 compared with control

It has been reported that cytokines such as IL-1β, IL-6, IL-8, IL-10, IL-11 and tumor necrosis factor (TNF-α) are important for the proliferation and malignancy of EC cells (Eritja et al., 2017). We tested the effects of si-YAP on the expression of these cytokines in both Ishikawa and AN3CA cells. qRT-PCR showed that si-YAP can significantly decrease the expression of IL-6 and IL-11 in Ishikawa cells (Fig. 3 a). Consistently, si-YAP can also decrease the expression of IL-6 and IL-11 in AN3CA cells (Fig. 3 b). si-YAP decreased the expression of IL-1β in AN3CA cells while not in Ishikawa cells, which indicated that the effects of YAP on IL-1β are cell line dependent. In Ishikawa cells, VP can decrease the expression of IL-6 and IL-11 via a concentration dependent manner (Fig. 3 c). The down regulation of IL-6 and IL-11 in Ishikawa cells treated with VP (Fig. 3 d) or transfected with si-YAP (Fig. 3 e) was confirmed by ELISA. Consistently, over expression of YAP (Fig. 3 f) can increase the expression of IL-6 and IL-11 in Ishikawa cells (Fig. 3 g). These results showed that YAP can regulate the expression of IL-6 and IL-11 in EC cells.

### Both IL-6 and IL-11 are involved in YAP regulated malignancy of EC cells

**Fig. 4:**
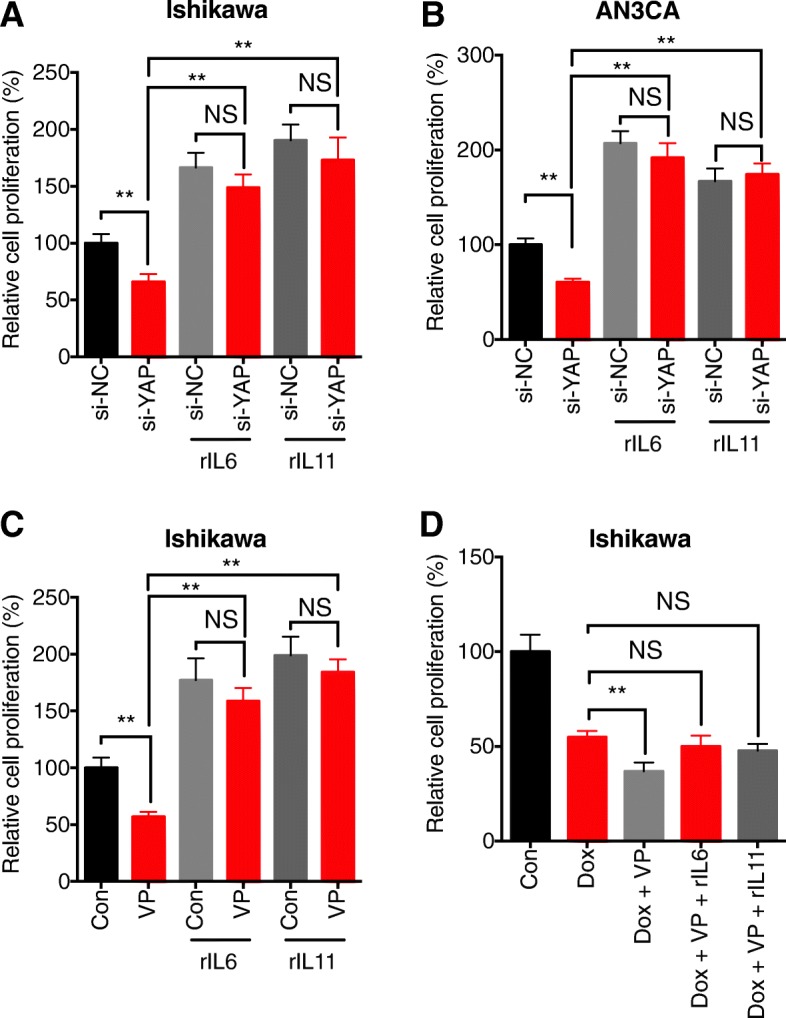
*Both IL-6 and IL-11 are involved in YAP regulated malignancy of EC cells****.*** After transfected with si-NC or si-YAP for 12 h, Ishikawa (**a**) or AN3CA (**b**) cells were further treated with rIL-6 (200 ng/ml) or rIL-11 (200 ng/ml) for 48 h. The cells proliferation was tested by CCK-8 kit; (**c**) After pre-treated with or without VP (5 μM) for 1 h, Ishikawa cells were further treated with rIL-6 (200 ng/ml) or rIL-11 (200 ng/ml) for 48 h; (**d**) After pretreated with or without Dox (1 μM) and/or VP (1 μM) for 1 h, Ishikawa cells were further treated with rIL-6 (200 ng/ml) or rIL-11 (200 ng/ml) for 48 h. Data are presented as means ± SD of three independent experiments. *p < 0.05, ** *p* < 0.01 compared with control

We then investigated whether IL-6 and IL-11 are involved in YAP regulated malignancy of EC cells. Our data showed that recombinant IL-6 (rIL-6) and rIL-11 can increase the proliferation of Ishikawa cells, further, both rIL-6 and rIL-11 can attenuate the si-YAP suppressed proliferation of Ishikawa cells (Fig. 4 a). Similar results were also observed in AN3CA (Fig. 4 b). Further, rIL-6 and rIL-11 also reversed the suppression effects of VP (5 μM) on the proliferation of Ishikawa cells (Fig. 4 c). In the presence of rIL-6 and rIL-11, 1 μM of VP increased Dox sensitivity of Ishikawa cells was also significantly reversed (Fig. 4 d). These data showed that both IL-6 and IL-11 are involved in YAP regulated malignancy of EC cells.

### YAP can directly regulate the transcription of IL-6, while not IL-11, in EC cells

**Fig. 5:**
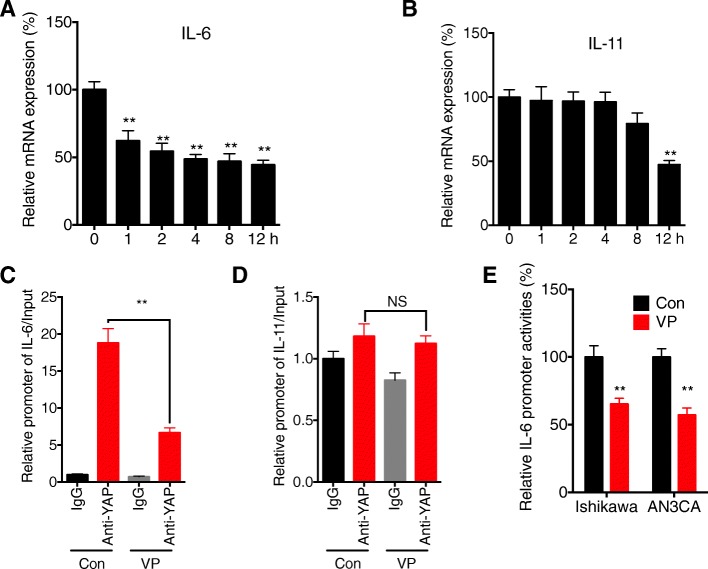
*YAP can directly regulate the transcription of IL-6, while not IL-11, in EC cells***.** Ishikawa cells were treated with 5 μM of VP for the indicated times, the expression of IL-6 (**a**) and IL-11 (**b**) was measured by use of qRT-PCR; Ishikawa cells were treated with or without VP for 1 h, the binding between YAP and promoter of IL-6 (**c**) or IL-11 (**d**) was measured by ChIP assay; (**e**) Ishikawa or AN3CA cells were treated with or without VP for 1 h, the luciferase activities of IL-6 promoter were measured by use of the dual-luciferase assay. Data are presented as means ± SD of three independent experiments. *p < 0.05, ** *p* < 0.01 compared with control

We further investigated the mechanisms involved in YAP regulated expression of IL-6 and IL-11 in EC cells. Our data showed that VP can rapidly decrease the expression of IL-6 in Ishikawa cells after treatment for 1 h (Fig. 5 a). However, VP can only decrease the expression of IL-11 in Ishikawa cells after treatment for more than 8 h (Fig. 5 b). We then investigated whether YAP can directly bind to the promoter of IL-6 or IL-11 by ChIP assay. Our data showed that YAP can directly bind to the promoter of IL-6, while VP can decrease the binding between YAP and promoter of IL-6 in Ishikawa cells (Fig. 5 c). However, there was no enrichment of promoter of IL-11 in YAP antibody as compared to that of IgG, further, VP had no effect on the binding efficiency (Fig. 5 d). The dual luciferase assay indicated that VP can suppress the promoter activity of IL-6 in both Ishikawa and AN3CA cells (Fig. 5 e). These results suggested that YAP can directly regulate the transcription of IL-6, while not IL-11, in EC cells.

### NF-κB is involved in YAP regulated transcription of IL-11 in EC cells

**Fig. 6:**
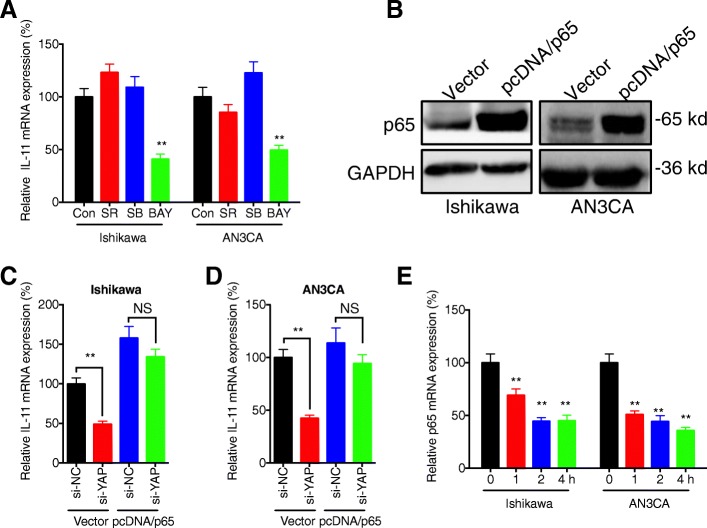
*NF-κB is involved in YAP regulated transcription of IL-11 in EC cells****.*** (**a**) Ishikawa or AN3CA cells were treated with or without inhibitor of AP-1 (SR 11302, 10 μM), SMAD2/3 (SB431542, 10 μM), and NF-κB (BAY 11–7082, 10 μM), respectively, for 24 h, the mRNA of IL-11 was measured by use of qRT-PCR; (**b**) Cells were transfected with vector control or pcDNA/p65 for 24 h, the expression of p65 was measured by western blot analysis; Ishikawa (**c**) or AN3CA (**d**) cells were pre-transfected with si-NC or si-YAP for 6 h, and then further transfected with vector control or pcDNA/p65 for 24 h; (**e**) Cells were treated with VP for the indicated times, the mRNA of p65 was measured by use of qRT-PCR. Data are presented as means ± SD of three independent experiments. ** *p* < 0.01 compared with control

IL-11 transcription is largely dependent upon transcription factors such as AP-1, SMADs, and p65NF-κB in cancer cells (Xu et al., 2016). EC cells were further treated with the inhibitors of AP-1 (SR 11302), SMAD2/3 (SB431542), and NF-κB (BAY 11–7082), respectively. Our data showed that only the inhibitor of NF-κB (BAY 11–7082) can significantly down regulate the mRNA expression of IL-11 in Ishikawa and AN3CAcells (Fig. 6a). Further, over expression of p65 (Fig. 6 b), one key component of NF-κB complex (Tak and Firestein, 2001), can reverse si-YAP induced down regulation of IL-11 in both Ishikawa (Fig. 6c) and AN3CA (Fig. 6d) cells. This might be due that the inhibitor of YAP can rapidly decrease the transcription of p65 in both Ishikawa and AN3CA cells (Fig. 6 e). Collectively, these results showed that NF-κB is involved in YAP regulated transcription of IL-11 in EC cells.

## Discussion

Although various studies indicated that YAP functions as an oncogene in most cancers (Zhao et al., 2010), the roles of YAP in the progression of EC remain unclear. Our present study revealed that the expression of YAP was upregulated in both EC cells and tissues as compared to their corresponding controls. Targeted inhibition of YAP by its siRNA or inhibitor can suppress the proliferation and increase the chemosensitivity of EC cells. Among the measured cytokines, YAP can regulate the expression of IL-6 and IL-11. While rIL-6 and rIL-11 can reverse YAP regulated proliferation and Dox sensitivity of EC cells. Mechanistically, YAP can directly bind with the promoter of IL-6 to increase its transcription. As to IL-11, the upregulation of p65 is involved in YAP regulated its expression. Collectively, our present study revealed that YAP can trigger the malignancy of EC cells via upregulation of IL-6 and IL-11.

Our study revealed that the upregulation of YAP in EC cells can trigger its proliferation and decrease its chemosensitivity. Tsujiura et al. (Tsujiura et al., 2014) reported that higher levels of nuclear YAP were associated with poor prognostic factors of EC patients. Consistently, YAP can promote the proliferation, anchorage independent growth, invasion and migration of human EC HEC-1-B cells (Tsujiura et al., 2014). The reasons responsible for upregulation of YAP in EC cells and tissues are currently unknown. It has been reported that YAP gene can be amplified in various cancers such as breast, esophageal, hepatocellular cancer, ependymoma, malignant mesothelioma and medulloblastoma (Overholtzer et al., 2006; Zender et al., 2006). However, comprehensive analyses of somatic alterations suggested that this amplification was not observed in EC cells and tissues (Getz et al., 2013). Therefor the mechanisms responsible for the upregulation of YAP in EC cells need further study.

Our data suggested that the upregulation of IL-6 and IL-11 was involved in YAP regulated proliferation and chemosensitivity of EC cells. YAP can induce the expression of IL-6 in hepatocellular carcinoma cells and then recruit tumor-associated macrophages (Zhou et al., 2018). Further, IL-6 has been proved as the transcriptional target of YAP involved in basal-like breast cancer (Kim et al., 2015). Our data confirmed that YAP can directly bind with the promoter of IL-6 to regulate its transcription in EC cells. In addition, our data also showed that YAP can regulate the expression of IL-11 in EC cells but not directly bind with its promoter. This might be due to that YAP can upregulate p65 induced transcription of IL-11. Both IL-6 and IL-11 can promote the malignancy of EC cells via triggering cell proliferation, migration and invasion (Chu et al., 2018; Lay et al., 2012; Yap et al., 2010).

## Conclusions

We demonstrated that the upregulation of YAP can increase the proliferation and decrease the chemosensitivity of EC cells via upregulation of IL-6 and IL-11. Although further mechanisms and in vivo evidences are needed, our data suggested that targeted inhibition of YAP might be a potential therapy approach for treatment of EC patients.

## Data Availability

All data and material are available.

## Abbreviations

ChIP: Chromatin immunoprecipitation
Dox: doxorubicin
EC: endometrial cancer
ECL: enhanced chemiluminescence
ELISA: Enzyme-linked immunoassays
ESC: endometrial stromal cell
HRP: horseradish peroxidase
ILs: interleukins
LATS1/2: large tumor suppressor 1/2
MST1/2: mammalian Ste20-like kinases 1/2
PVDF: polyvinylidene fluoride
SD: standard deviation
TNF: tumor necrosis factor
VP: verteporfin
YAP: Yes-associated protein
